# Supplementary material for: Plasma Fibulin-5 as a Novel Marker for Advanced Fibrosis in Chronic Hepatitis C
Source: Gastro Hep Adv. 2025 Oct 10;5(2):100827. doi: 10.1016/j.gastha.2025.100827 (PMC12681702; doi:10.1016/j.gastha.2025.100827)

Supplementary Material & methods

Plasma fibulin-5 as a novel marker for advanced fibrosis in chronic hepatitis C

Yutaka Yasui, Misako Sato-Matsubara, Masaru Enomoto, Tsutomu Matsubara, Mana Kosugi, Kirara Inoue, Truong Huu Hoang, Hideto Yuasa, Hideki Fujii, Atsuko Daikoku, Yoshihiro Ikura, Etsushi Kawamura, Sawako Uchida-Kobayashi, Akihiro Tamori, Norifumi Kawada

Table of Contents

Fig. S1 Study flowchart
IHC: immunohistochemistry

Fig. S2 Fibulin-5 (FBLN5)-positive areas were significantly correlated with fibers quantified by azan staining and elastic fibers quantified by orcein staining

Fig. S3 The protein expression of human hepatic stellate cell (HHSteC) and human dermal fibroblast in response to transforming growth factor (TGF) β-1.

Fig. S4 The HHSteC culture medium was analyzed to confirm whether FBLN5 was secreted from activated HSCs. The Coomassie Brilliant Blue staining confirmed that the amount of protein in the medium samples was consistent across all samples, indicating equal protein loading.

Table S1. The list of primers for real-time PCR

Table S2. Multivariate analysis for the factors associated with F3–F4 stage

Table S1. The list of primers for real-time PCR

Gene	Forward (5'→3')	Reverse (5'→3')
h18S	AGTCCCTGCCCTTTGTACACA	CGATCCGAGGGCCTCACTA
hELN	GCAGGAGTTAAGCCCAAGG	TGTAGGGCAGTCCATAGCCA
hFBLN5	CTCACTGTTACCATTCTGGCTC	GACTGGCGATCCAGGTCAAAG
hFBN1	TTTAGCGTCCTACACGAGCC	CCATCCAGGGCAACAGTAAGC
hMFAP4	TACCAGTCAGACGGCGTGTA	CCACTCGCAGCTCATACTTCT

PCR, polymerase chain reaction

Table S2. **Multivariate analysis for the factors associated with F3–F4 stage**

	Univariate analysis			Multivariate analysis		
Factors	Odds ratio	95% C.I.	P value	Odds ratio	95% C.I.	P value
Age; per 1-year increase	1.05	0.99–1.11	0.10			
Sex; male	3.44	1.12–10.5	0.03			
Platelet; per 1×10^4(/μl) increase	0.78	0.67–0.90	0.0009			
AST; per 1 (U/L) increase	1.01	1.00–1.01	0.06			
ALT; per 1 (U/L) increase	1.01	0.99–1.01	0.11			
Collagen type IV; per 100 (ng/ml) increase	1.92	1.31–2.81	0.0008	1.37	1.11–1.70	0.004
FBLN5; per 100 (ng/ml) increase	1.28	1.11–1.48	0.0007	2.33	1.41–3.88	0.001

Fig. S1

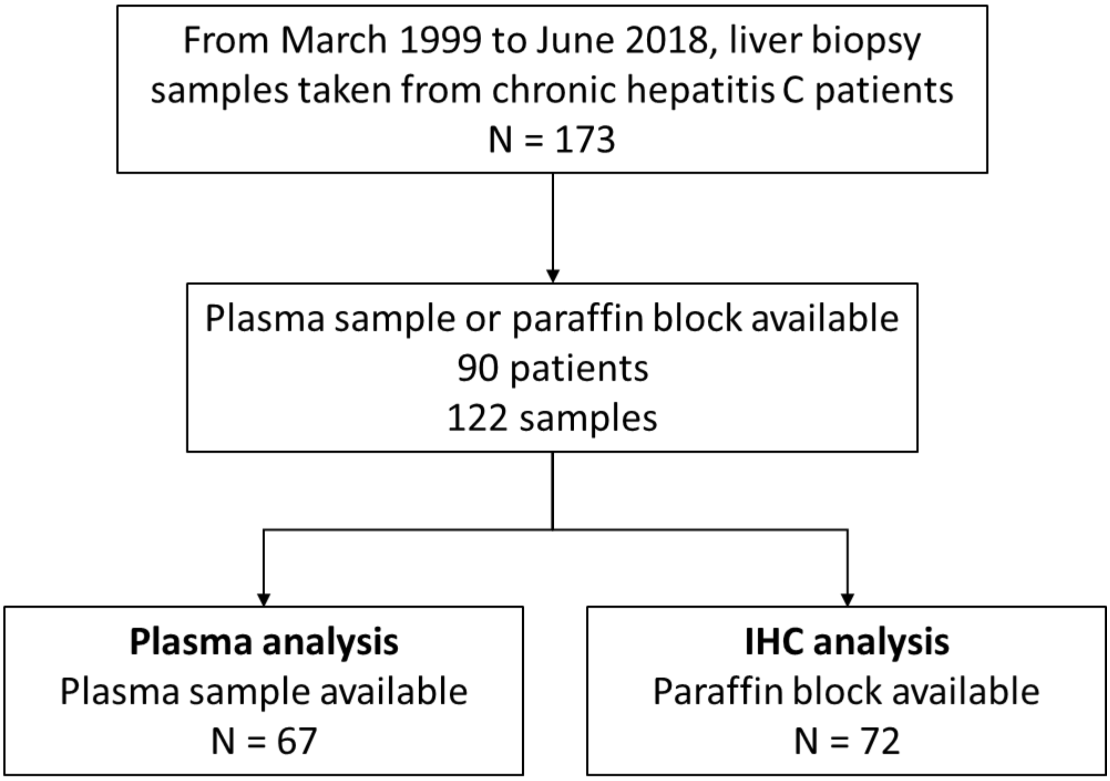
From March 1999 to June 2018, liver biopsy samples taken from chronic hepatitis C patients
N = 173
Plasma sample or paraffin block available
90 patients
122 samples
Plasma analysis
Plasma sample available
N = 67
IHC analysis
Paraffin block available
N = 72

Fig. S2

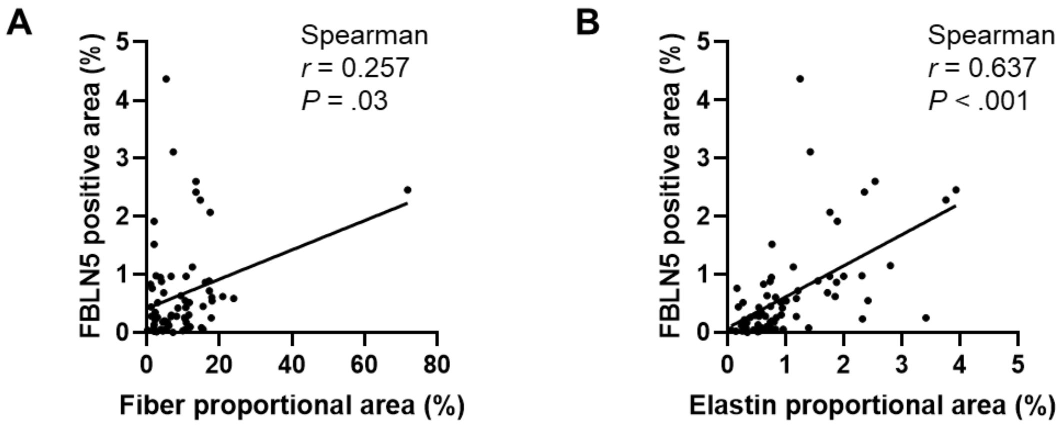
A
Spearman
r = 0.257
P = .03
FBLN5 positive area (%)
Fiber proportional area (%)
0
1
2
3
4
5
0
20
40
60
80
B
Spearman
r = 0.637
P < .001
FBLN5 positive area (%)
Elastin proportional area (%)
0
1
2
3
4
5
0
1
2
3
4
5

Fig. S3

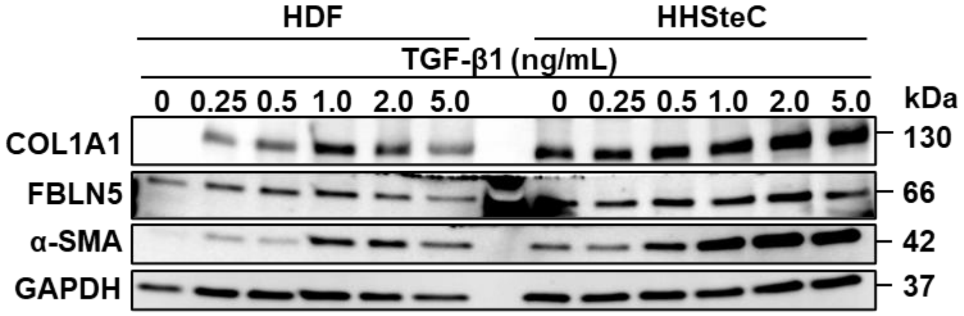

34

Fig. S4

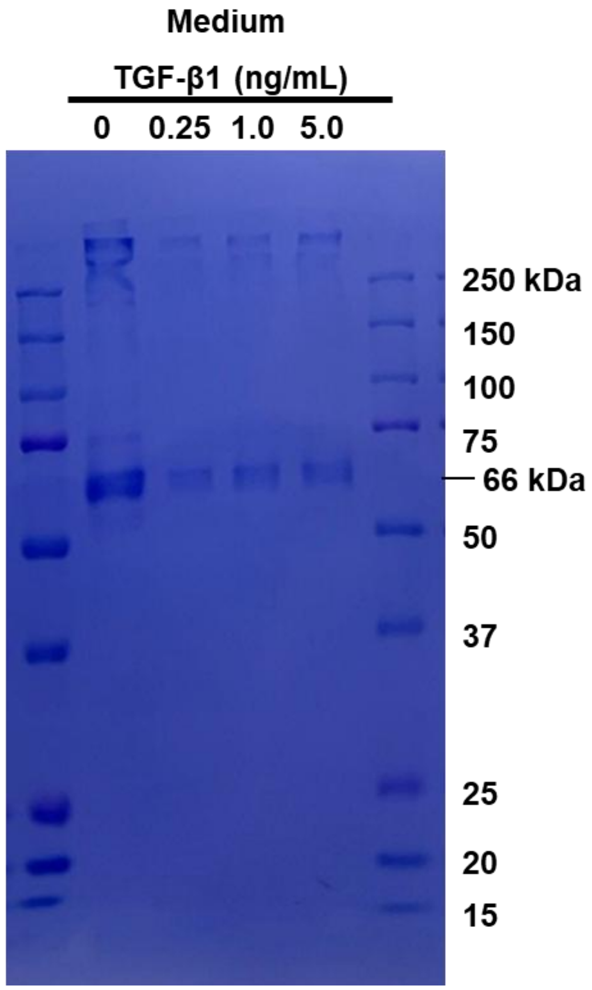

Supplement: Supplementary Material & Methods [file mmc1.pdf]
