# Supplementary material for: Plasma Fibulin-5 as a Novel Marker for Advanced Fibrosis in Chronic Hepatitis C
Source: Gastro Hep Adv. 2025 Oct 10;5(2):100827. doi: 10.1016/j.gastha.2025.100827 (PMC12681702; doi:10.1016/j.gastha.2025.100827)

Gastro Hep Advances 2026;5:100827

ORIGINAL RESEARCH—CLINICAL

Plasma Fibulin-5 as a Novel Marker for Advanced Fibrosis in Chronic Hepatitis C

Yutaka Yasui,[1,2,*] Misako Sato-Matsubara,[1,3,*] Masaru Enomoto,[1] Tsutomu Matsubara,[4] Mana Kosugi,[4] Kirara Inoue,[1,5] Truong Huu Hoang,[6,7] Hideto Yuasa,[4] Hideki Fujii,[1] Atsuko Daikoku,[4] Yoshihiro Ikura,[8] Etsushi Kawamura,[1] Sawako Uchida-Kobayashi,[1] Akihiro Tamori,[1,9] and Norifumi Kawada[1]

[1]*Department of Hepatology, Graduate School of Medicine, Osaka Metropolitan University, Osaka, Japan;* [2]*Department of Gastroenterology and Hepatology, Musashino Red Cross Hospital, Tokyo, Japan;* [3]*Laboratory of Cellular and Molecular Biology, Graduate School of Veterinary Science, Osaka Metropolitan University, Osaka, Japan;* [4]*Department of Anatomy and Regenerative Biology, Graduate School of Medicine, Osaka Metropolitan University, Osaka, Japan;* [5]*Department of Biology, Graduate School of Science, Osaka Metropolitan University, Osaka, Japan;* [6]*Centre for Genetic Consultation and Cancer Screening, 108 Military Central Hospital, Hanoi, Vietnam;* [7]*Vietnamese German Center for Medical Research (VG-CARE), 108 Military Central Hospital, Hanoi, Vietnam;* [8]*Department of Pathology, Takatsuki General Hospital, Takatsuki, Japan; and* [9]*Department of Gastroenterology, Kashiwara Municipal Hospital, Osaka, Japan*

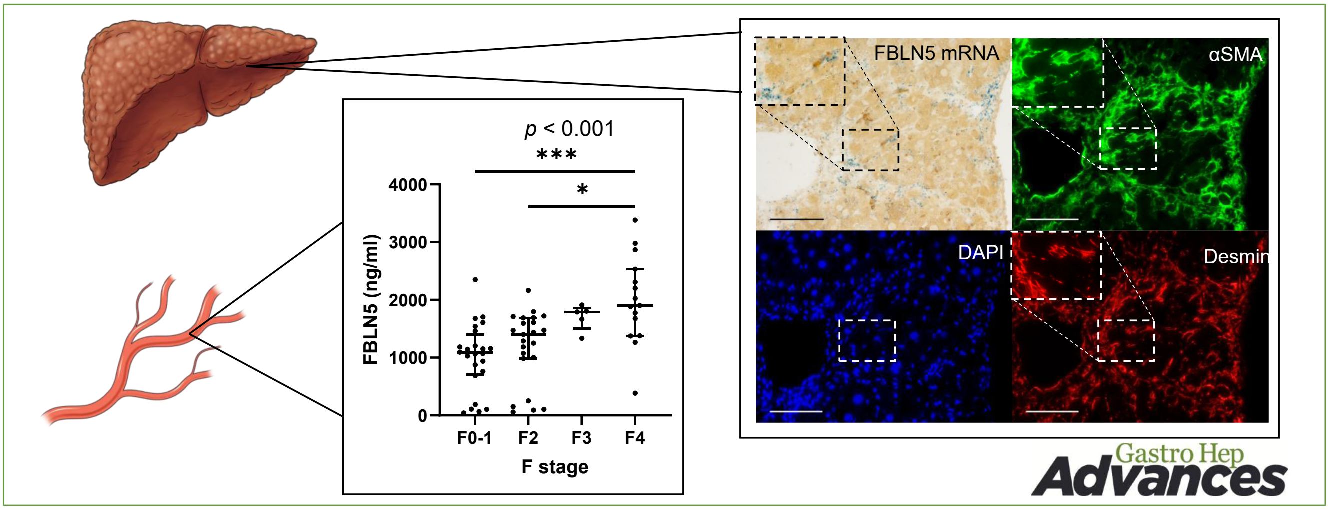

BACKGROUND AND AIMS: Despite remarkable advances in the treatment of viral hepatitis, liver fibrosis sometimes persists after viral eradication. The accumulation of elastic fiber is associated with unfavorable clinical outcomes. We aimed to clarify the utility of plasma fibulin-5 (FBLN5), a component of elastic fibers, in assessing liver fibrosis in patients with chronic hepatitis. **METHODS:** We reviewed 90 patients with chronic hepatitis C who underwent liver biopsy. Using immunohistochemistry and in situ hybridization, we investigated the localization of FBLN5 and its correlation with α-smooth muscle actin (αSMA). We then analyzed mRNA and protein expression in human hepatic stellate cells (HHSteCs) and primary mouse stellate cells. Lastly, we evaluated the utility of plasma FBLN5 for predicting liver fibrosis in patients with hepatitis C. **RESULTS:** Immunohistochemical analysis revealed a correlation between the quantity of FBLN5 and αSMA ($r = 0.65$) as well as FBLN5 and fibrosis stage ($r = 0.30$). FBLN5 was localized to areas enriched for αSMA-positive cells. In situ hybridization confirmed the colocalization of FBLN5 mRNA with αSMA and desmin-positive cells. Single nuclear RNA-sequencing analysis revealed that FBLN5 expression is predominantly expressed in the hepatic stellate cell/fibroblast cluster in other etiology, and the expression level was higher in cirrhotic livers. FBLN5 was overexpressed in cultured HHSteCs

*These authors equally contributed to this work.

Abbreviations used in this paper: αSMA, α-smooth muscle actin; AST, aspartate aminotransferase; AUROC, area under the receiver-operating characteristic; FBLN5, fibulin-5; FIB-4, fibrosis-4; HDF, human dermal fibroblast; HHSteC, human hepatic stellate cells; HSCs, hepatic stellate cells; IHC, immunohistochemistry; snRNA-seq, single nuclear RNA-sequencing; TGF, transforming growth factor; UMAP, uniform manifold approximation and projection.

Most current article

Copyright © 2025 The Authors. Published by Elsevier Inc. on behalf of the AGA Institute. This is an open access article under the CC BY license (http://creativecommons.org/licenses/by/4.0/).
2772-5723
https://doi.org/10.1016/j.gastha.2025.100827

in response to 2.0 μg/mL transforming growth factor-β1 ($P < .001$), with increased FBLN5 concentration in the medium of transforming growth factor-β1-treated activated HHSteC. FBLN5 upregulation was also confirmed in primary-cultured mouse stellate cells. Finally, plasma FBLN5 levels increased with fibrosis progression in patients with chronic hepatitis C ($P < .001$). **CONCLUSION:** FBLN5 may represent the activation of hepatic stellate cells, and plasma FBLN5 levels have the potential to predict advanced fibrosis, especially in fibrosis related to elastic fibers.

Keywords: Liver Fibrosis; Elastic Fiber; Cirrhosis; Activated Hepatic Stellate Cells

Introduction

During the past decade, remarkable improvements have been made in the treatment of chronic hepatitis C.[1] However, the complete elimination of hepatitis C is still a distant goal.[2] Even after viral eradication, liver-related complications, including hepatocellular carcinoma, continue to affect patients with advanced fibrosis, making them a global healthcare issue.[3] In addition, the reversibility of severe liver fibrosis remains unclear.[4–7] A key factor in liver fibrosis is the remodeling of the extracellular matrix, particularly the cross-linking of collagen and elastic fibers, which hinders fibrosis regression.[8] Notably, recent studies suggest that elastic fibers degrade more slowly than collagen fibers.[9] Our research, along with others, has shown that the accumulation of elastic fiber is associated with unfavorable outcomes including the development of hepatocellular carcinoma in patients with chronic hepatitis C.[10,11] Thus, the amount of elastic fibers is important information, not only for the regressive status of fibrosis but also for the clinical course of patients with advanced fibrosis. Currently, noninvasive methods, such as serum[12–15] and imaging[16–18] markers, are commonly used to detect advanced fibrosis. However, factors such as inflammation, age, or sex affect diagnostic accuracy. There remains an unmet need to assess post-treatment changes in fibrosis, including fiber quality.

Collagen fibers are the primary component of the extracellular matrix, whereas elastic fibers appear in the advanced stages of fibrosis.[19,20] A mature elastic fiber is composed of a core of tropoelastin and fibulin-5 (FBLN5), surrounded by microfibrillar proteins including fibrillin-1 and microfibrillar-associated protein-4.[21] Among these, FBLN5 is crucial for the assembly of elastic fibers into mature and organized structures.[22–24] FBLN5 consists of 367 amino acids and contains an epidermal growth factor-like domain, which binds to αvβ3, αvβ5, and α9β1 integrin on the cell surface.[25,26] Nakamura et al initially reported that FBLN5 is essential for arterial development.[22] Proteomics analysis revealed that liver FBLN5 expression correlates with the degree of liver fibrosis.[27] However, little is known about peripheral FBLN5 in patients with chronic liver disease. Therefore, we aimed to evaluate the usefulness of peripheral FBLN5 as a potential biomarker for liver fibrosis.

In this study, we first examined FBLN5 levels in human liver biopsy samples. In addition, we performed an *in vitro* experiment to explore the mechanism underlying increased FBLN5 expression. Finally, we investigated whether plasma FBLN5, a noninvasive marker, could predict the degree of liver fibrosis in patients with chronic hepatitis C.

Materials and Methods

Patients

A total of 90 patients with chronic hepatitis C who underwent liver biopsy were retrospectively identified from our clinical database. Data on sex (male/female) were collected from medical records. Gender identity was not available in this study. Both male and female patients were eligible, and no exclusion criteria were based on sex. The sex distribution of enrolled patients was reported to ensure adequate representation. Biochemical data were collected from the database, and the fibrosis-4 (FIB-4) index[14] and the aspartate aminotransferase (AST) to platelet ratio index[12] were calculated by the following formulas: FIB-4 index $=$ age (years) $\times$ AST [U/L]/(platelet count [10^9/L] $\times$ $\sqrt{\text{alanine}}$ aminotransferase [U/L], AST to platelet ratio index $=$ (AST [U/L]/upper limit of normal of AST [U/L]) $\times$ 100/platelet count [10^9/L]. Among liver biopsy samples taken from patients with chronic hepatitis C (Supplementary Material & Methods Figure A1), paraffin blocks were available from 72 patients, for which we performed immunohistochemistry (IHC) for FBLN5 (IHC analysis cohort). We also analyzed plasma samples from 67 participants (plasma sample cohort). Plasma samples were stored at −80 °C until measurement by enzyme-linked immunosorbent assay (ELISA). Liver biopsy specimens were sliced 4 μm thick and stained with hematoxylin and eosin, azan, and orcein staining to evaluate the histological grade as well as fiber quantity. An experienced pathologist blinded to the clinical data diagnosed the fibrosis stage (F0–F4 stage) and activity grade (A0–A3 grades) according to the METAVIR scoring system.[28,29] Written informed consent was obtained from all patients. This study was conducted in accordance with the Declaration of Helsinki and approved by the Ethics Committee of Osaka Metropolitan University Graduate School of Medicine (approval number 1646).

Fiber Quantification Using Azan-Stained Sections

Digital images of azan-stained liver biopsy specimens were captured using a ×20 lens by BZ-X800 (Keyence, Osaka, Japan), followed by fiber quantification using BZ-X800 analyzing software. Quantification of the proportional fiber area was performed by (i) distinguishing fibers based on differences in hue and (ii) calculating the fiber mass as a ratio to the area of the entire biopsy specimen.

IHC Staining

IHC was performed to assess the quantity of FBLN5 and α-smooth muscle actin (αSMA), a biomarker of activated hepatic stellate cells (HSCs), in the liver. Paraffin-embedded samples

were sliced 4 μm thick and stained using rabbit polyclonal anti-elastin antibody 1:100 (ab21610, Abcam, Cambridge, UK), mouse monoclonal anti-αSMA antibody 1:300 (Clone 1A4, Dako, Agilent Technologies, Santa Clara, CA, USA), rabbit polyclonal anti-desmin antibody 1:100 (PA5-85187, Thermo Fisher Scientific), and rabbit polyclonal anti-FBLN5 antibody 1:100 (GTX64424, Genetex, Irvine, CA, USA). The quantity of the positive area was determined in the same way as described for fiber quantification.

In Situ Hybridization

In situ hybridization was performed according to a modified version of the manufacturer's protocol (ViewRNA ISH Tissue Assay, Thermo Fisher Scientific). In brief, 5 μm formalin-fixed paraffin-embedded tissue samples were sectioned, and in situ hybridization was performed. Second, the mouse FBLN5 probe (ViewRNA Type 6 probe set) was detected with alkaline phosphatase-conjugated anti-digoxigenin antibody and stained with ViewRNA-Fast Blue substrate. Finally, the sections were restained with anti-desmin antibody and an αSMA antibody for in situ hybridization staining.

ELISA Assay of FBLN5

Plasma and medium FBLN5 concentrations were assessed by ELISA (Immuno-biological Laboratories Co, Ltd) with the standard curve range of 0.08–5.0 ng/mL, according to the manufacturer's protocol.

Cell Culture

Human hepatic stellate cell (HHSteC) was purchased from ScienCell and maintained in complete medium following the manufacturer's recommendation (stellate cell medium supplemented with 10% fetal bovine serum, 1% stellate cell supplement, 100 units of penicillin, and 100 μg/mL of streptomycin solution). HHSteC was sub-confluently passaged and used for experiments between passages 6–13. LX-2 cells were gifted from Dr Scott Friedman (Mount Sinai Hospital). Human dermal fibroblasts (HDFs) were gifted from Dr Katsutoshi Yoshizato (Osaka Metropolitan University). HDF and LX-2 cells were maintained in Dulbecco's modified Eagle's medium supplemented with 10% fetal bovine serum. To assess gene and protein expression in the activated phenotype of HSCs or fibroblasts, recombinant transforming growth factor (TGF) β-1 was used with a typical concentration of 0.2 ng/mL, unless stated differently elsewhere. Recombinant human fibroblast growth factor 2 was purchased from Wako Pure Chemical Industries Ltd (Osaka, Japan), and platelet-derived growth factor-BB was purchased from PeproTech Inc (Rocky Hill, NJ, USA). The optimal concentrations of fibroblast growth factor 2 (4 ng/mL) and platelet-derived growth factor (20 ng/mL) were determined in our previous study.[30]

We assessed FBLN5 expression in primary mouse HSCs. Primary mouse HSCs were isolated from C57BL/6J mice (Japan SLC, Inc, Shizuoka, Japan) in our laboratory, using a previously described method.[31]

Real-Time PCR

mRNA expression was determined using quantitative real-time PCR (Bio-Rad, Hercules, CA, USA). Cultured cells were lysed in TRIZOL reagent (Thermo Fisher Scientific, Waltham, MA, USA) and purified by the Direct-zol RNA miniPrep kit (Zymo Research, Irvine, CA, USA). Complementary DNA was generated using SuperScript III reverse transcriptase (Thermo Fisher Scientific). The primers used for PCR are listed in Supplementary Material & Methods, Table A1. The relative expression level was normalized to 18S expression, and fold change in expression was calculated using the $2^{-\Delta\Delta CT}$ method.

Western Blot Analyses

Cultured cells were lysed in a radioimmunoprecipitation assay buffer containing protease inhibitors (cOmplete Mini, Roche Applied Science, Basel, Switzerland). After determining the protein concentration using bicinchoninic acid assay, the cell lysate was diluted in distilled water and an SDS sample buffer to equalize the concentration. Proteins were separated by sodium dodecyl sulfate–polyacrylamide gel electrophoresis into 10% polyacrylamide gels (DRC, Tokyo, Japan) and transferred to 0.45 μm polyvinylidene difluoride membranes (Bio-Rad). The membranes were incubated overnight with primary antibodies. The primary antibodies used in this study were anti-FBLN5 antibody (1:1,000, rabbit polyclonal, GTX64424, Genetex, Irvine, CA, USA) and anti-elastin antibody (1:1,000, mouse monoclonal, sc-58756, Santa Cruz Biotechnology, Dallas, TX, USA). The secondary antibodies used were horseradish peroxidase-conjugated goat anti-mouse or anti-rabbit antibodies (1:4,000, Dako).

Single Nuclear RNA-Sequencing Analysis With the Data Sets From the Gene Expression Omnibus

We investigated cell-specific expression of FBLN5 through single nuclear RNA-sequencing (snRNA-seq) analysis of liver cells to explore its significance in other etiology. The snRNA-seq analysis was carried out with data set (GSE185477 [2 normal livers], GSE174748 [2 normal livers and 2 livers with steatotic liver disease], and GSE212046 [2 cirrhotic livers with steatohepatitis]) that were gain from gene expression omnibus. The investigation was performed by the R statistical software (v.4.3.3) and Seurat package (v.5.0.3) after the data set was changed to Seurat object. The Seurat objects were integrated using the anchor-based robust principal component analysis integration. Filtering was applied to remove any cells with greater than 15% mitochondrial genes, less than 200 total genes, or more than 5000 total genes. Cell types were annotated based on the expression of specific gene markers.

HSCs/Fibroblasts: *DCN*, *ACTA2*, *COL3A4*.
B cells: *MS4A1*, *BANK1*, *CD37*.
Plasma B cells: *JCHAIN*, *MZB1*, *IGKC*.
Cholangiocytes: *KRT7*, *ANXA4*, *CFTR*.
NK/T cells: *CD2*, *CD247*, *IL7R*
Macrophages: *CD163*, *MARCO*, *C1QA*.
LSECs (Liver sinusoidal endothelial cells): *STAB2*, *FCN3*, *VWF*
Hepatocytes: *CYP3A4*, *CYP2E1*, *FABP1*.

Then, uniform manifold approximation and projection (UMAP) and violin plots were generated.

Statistical Analysis

Continuous variables with normal distribution were presented as means $\pm$ SD, while those without were presented as

Table 1. Baseline Characteristics of the Patients Included in This Study

Factor	Group	N = 90
Sex, n (%)	Female	45 (50.0)
	Male	45 (50.0)
Age (y)		64 [54, 70]
Platelet ($10^3/\mu L$)		155 [121, 196]
ALT (U/L)		28 [17, 66]
GGT (IU/L)		36 [18, 78]
Fibrosis stage, n (%)	0–1	37 (41.1)
	2	26 (28.8)
	3	11 (12.2)
	4	16 (17.8)

Data are shown as number (%) or median (IQR).
ALT, alanine aminotransferase; GGT, gamma-glutamyl transferase.

medians and ranges. The difference between 2 variables was analyzed using either the Student's *t*-test or the Mann–Whitney U test in accordance with its distribution. To compare three or more variables, Kruskal–Wallis analysis was performed, followed by Bonferroni post hoc analysis. *P* values < 0.05 were considered statistically significant. All statistical analyses were performed using EZR software (Saitama Medical Center, Jichi Medical University, Saitama, Japan), a graphical interface version of R statistics (The R Foundation for Statistical Computing, Vienna, Austria),[32] or GraphPad PRISM (version 7.04, GraphPad Software, San Diego, CA, USA).

Results

Baseline Characteristics of Patients Included in This Study

A total of 90 patients with chronic hepatitis C were enrolled in this study. All patients underwent liver biopsy either before or after antiviral treatment. We assessed liver FBLN5 IHC in 72 patients and peripheral FBLN5 in 67 patients (Supplementary Material & Methods Figure A1). Table shows the baseline characteristics of the patients included in this study.

FBLN5 Expression Correlated With αSMA in Advances With Liver Fibrosis

First, we compared the liver fibrosis stage and FBLN5. In IHC of FBLN5 in 72 liver biopsy samples, the FBLN5-positive area significantly increased in correlation with the liver fibrosis stage (Figure 1A). This correlation was confirmed by quantifying the fiber-proportional area in the biopsy specimens. FBLN5-positive areas were significantly correlated with fibers quantified by azan staining for collagen fibers and orcein staining for elastic fibers (Supplementary Material and methods Figure A2). Similarly, the αSMA positive area showed a significant increase in line with advances of liver fibrosis stage (Figure 1B). The FBLN5-and αSMA-positive areas were strongly correlated (Spearman $r = 0.654$; $P < .001$) (Figure 1C). Based on these results, we assessed the localization of FBLN5 and αSMA in immunofluorescence-stained specimens. FBLN5 expression was abundantly increased in F4-stage cirrhotic livers compared to that in F1-stage livers and was localized to areas enriched for αSMA-positive cells (Figure 1D). Furthermore, in situ hybridization assays using mirror section tissue confirmed that FBLN5 mRNA colocalized with αSMA and desmin-positive cells. This suggests that FBLN5 was produced by activated HSCs (Figure 1E).

Cell-Specific Expression of FBLN5 in Liver Identified by snRNA-Seq

snRNA-seq analysis using datasets from the gene expression omnibus identified several cell clusters on the UMAP plot based on specific gene markers (Figure 2A). The cell-specific expression of FBLN5 in human liver samples revealed that FBLN5 expression is predominantly expressed in the HSC/fibroblast cluster (Figure 2B). FBLN5-expressing cells were distributed within the HSC/fibroblast cluster on the UMAP (Figure 2C), and the expression level was higher in cirrhotic livers with steatohepatitis compared to steatotic liver disease or normal livers (Figure 2D).

Gene and Protein Expression of FBLN5 Upregulated by TGF-β1 in HSCs

Next, we investigated gene expression in HHSteC and LX-2 cells (human HSCs). Among 4 major elastic fiber components, elastin (30.2 ± 4.2-fold change; $P < .001$) and FBLN5 (3.1 ± 0.1-fold change, $P < .001$) were significantly upregulated in response to TGF-β1, whereas microfibrillar associated protein-4 and fibrillin-1 did not change after TGF-β1 stimulation in HHSteC (Figure 3A). FIBLN5 expression increased in a dose-dependent manner (Figure 3B). The up-regulation of FBLN5 by TGF-β1 was also observed in LX-2. Next, we investigated HHSteC protein expression in response to major growth factors.[30] Among TGF-β1, platelet-derived growth factor, and fibroblast growth factor 2, only TGF-β1 increased FBLN5 expression in addition to αSMA and collagen expressions (Figure 3C). The FBLN5 protein expression of HHSteC was increased by TGF-β1 in a dose-dependent manner, which was also seen in HDF. The dose-dependence of FBLN5 protein expression by TGF-β1 was more pronounced in HHSteC than in HDF (Figure 3D, Supplementary Material & Methods Figure A3). We then analyzed the HHSteC culture medium to confirm whether FBLN5 was secreted from activated HSCs. The level of FBLN5 protein in culture medium increased in response to TGF-β1 in a dose-dependent manner (Figure 3E). Coomassie Brilliant Blue staining confirmed that the amount of protein in the medium samples was consistent across all samples, indicating equal protein loading (Supplementary Material & Methods

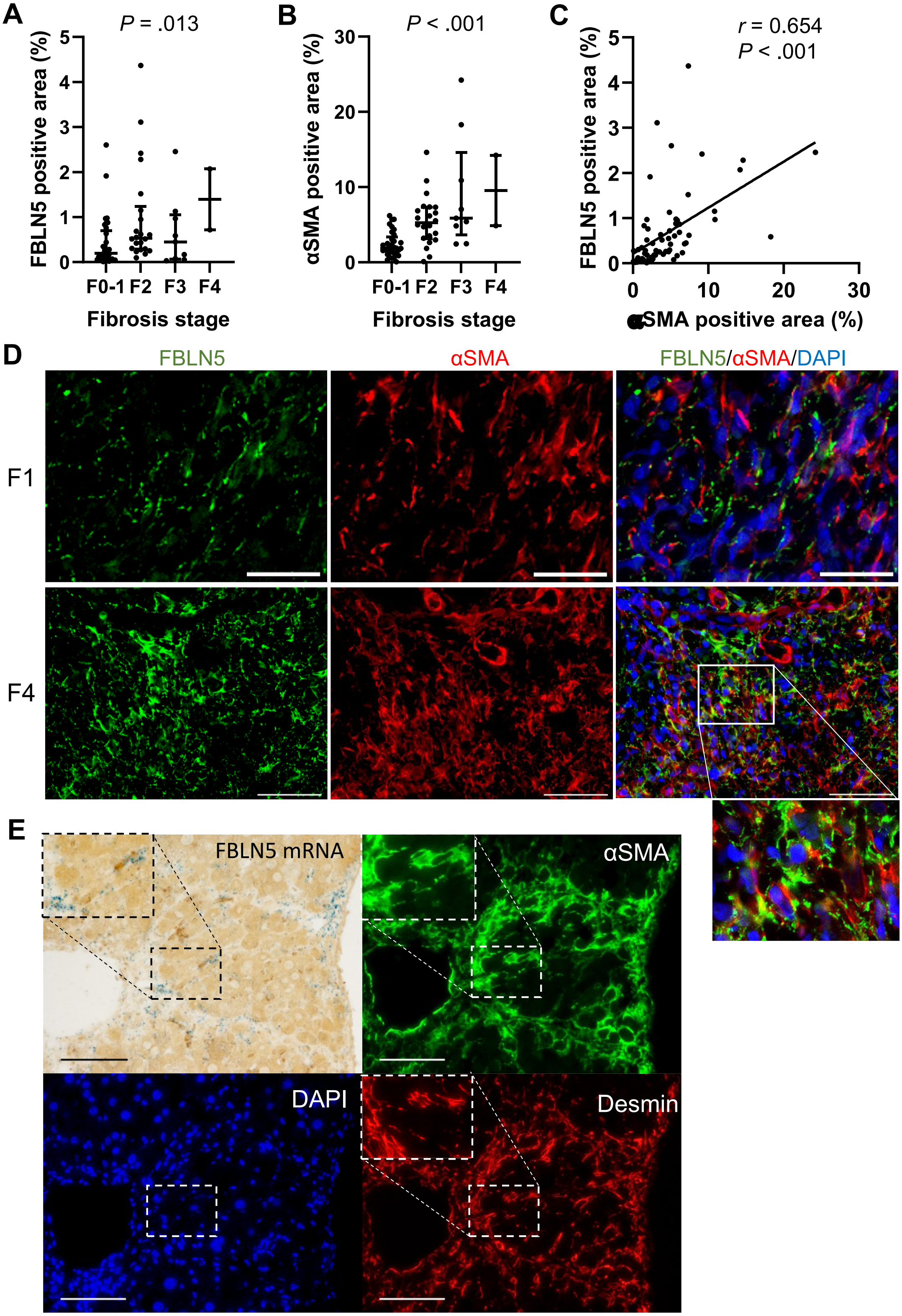

Figure 1. Correlation of FBLN5 expression in αSMA-positive cells. In the human liver biopsy specimen, the FBLN5-positive area (A) as well as the α-smooth muscle actin (αSMA)-positive area (B) correlates with fibrosis stage. The FBLN5-positive area significantly correlates with αSMA-positive area (C). Immunohistochemistry reveals that FBLN5 expression increases in F4-stage compared to F1-stage and localizes to areas enriched for αSMA-positive cells (D). In situ hybridization assays confirm that FBLN5 mRNA colocalizes with αSMA- and desmin-positive cells (E). DAPI, 4',6-diamidino-2-phenylindole.

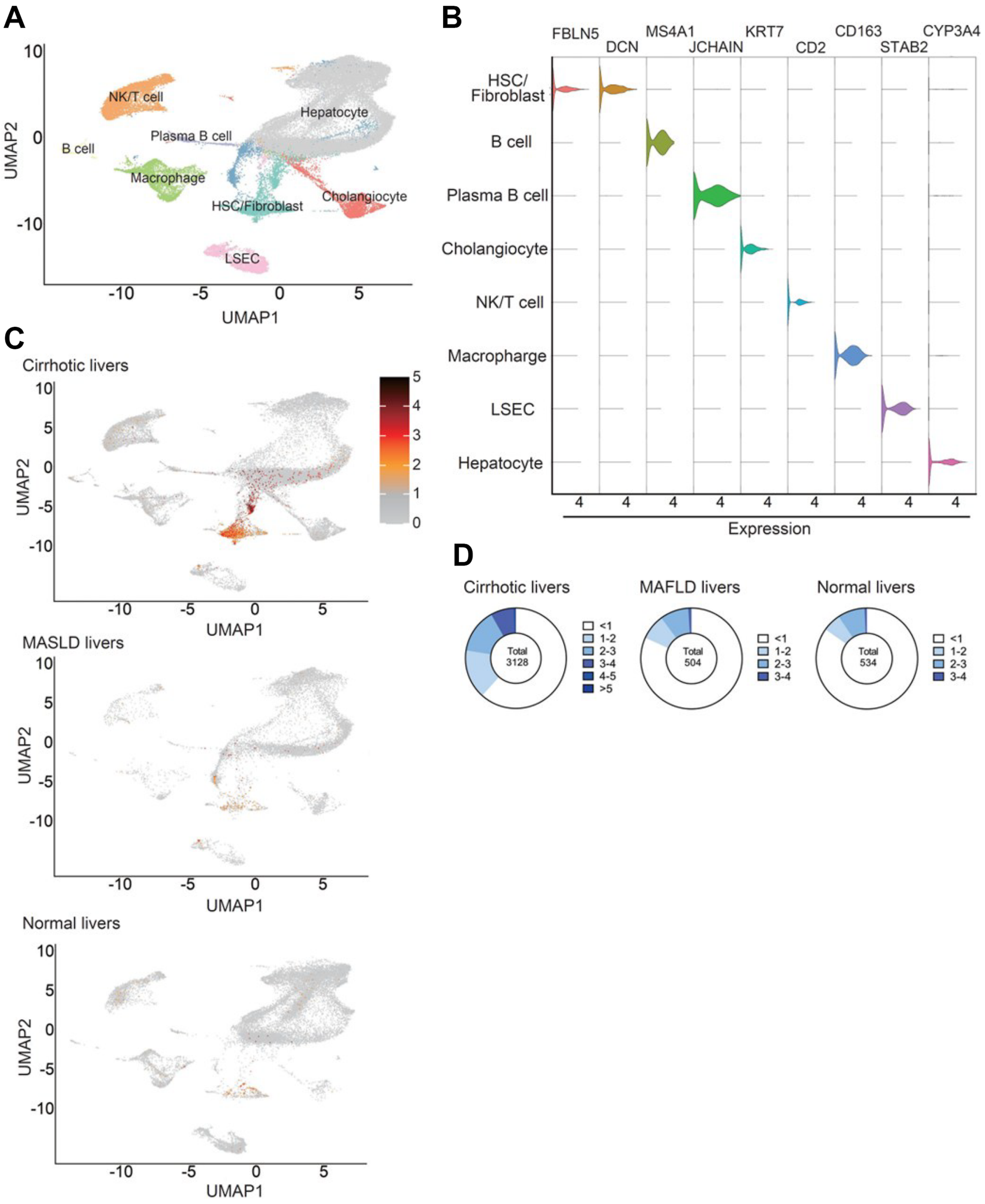

Figure 2. Human liver FIBLN5 exhibits specificity for HSCs/fibroblasts (A) UMAP plot. Cell type was determined with specific gene makers, as described in Materials and Methods. (B) Violin plot of the cell-specific FBLN5 expression. (C) FBLN5-expressing cells on the UMAP of cirrhotic, steatotic liver disease, and normal livers. (D) FBLN5 level and cell number in the HSC/fibroblast cell population. DVCN, decorin; MAFLD, metabolic dysfunction-associated fatty liver disease; MASLD, metabolic dysfunction-associated steatotic liver disease; STAB2, stabilin-2.

Figure A4). TGF-β1-induced FBLN5 expression in the medium was also confirmed by ELISA (Figure 3F).

Upregulation of FBLN5 in Spontaneously Activated Mouse Primary HSCs

Furthermore, we observed FBLN5 expression in primary mouse HSCs. Primary mouse HSCs cultured in plastic dishes have been reported to be activated spontaneously.[33] FBLN5 mRNA expression was significantly upregulated after 24 hours of culture in a plastic dish. Upregulation of collagen 1α1 and αSMA demonstrated spontaneous activation in the same sample (Figures 4A–C). To evaluate the secreted proteins, we also analyzed their concentration in the culture medium supernatant. FBLN5 concentration in the medium increased in a time-dependent manner (Figure 4D).

Plasma FBLN5 Correlated With Fibrosis Stage

We then investigated the plasma level of FBLN5 in 67 patients with chronic hepatitis C. Depending on the fibrosis stage, plasma FBLN5 levels increased with disease progression ($P < .001$, Figure 5A), whereas plasma FBLN5 levels did not increase in association with activity grade (data not

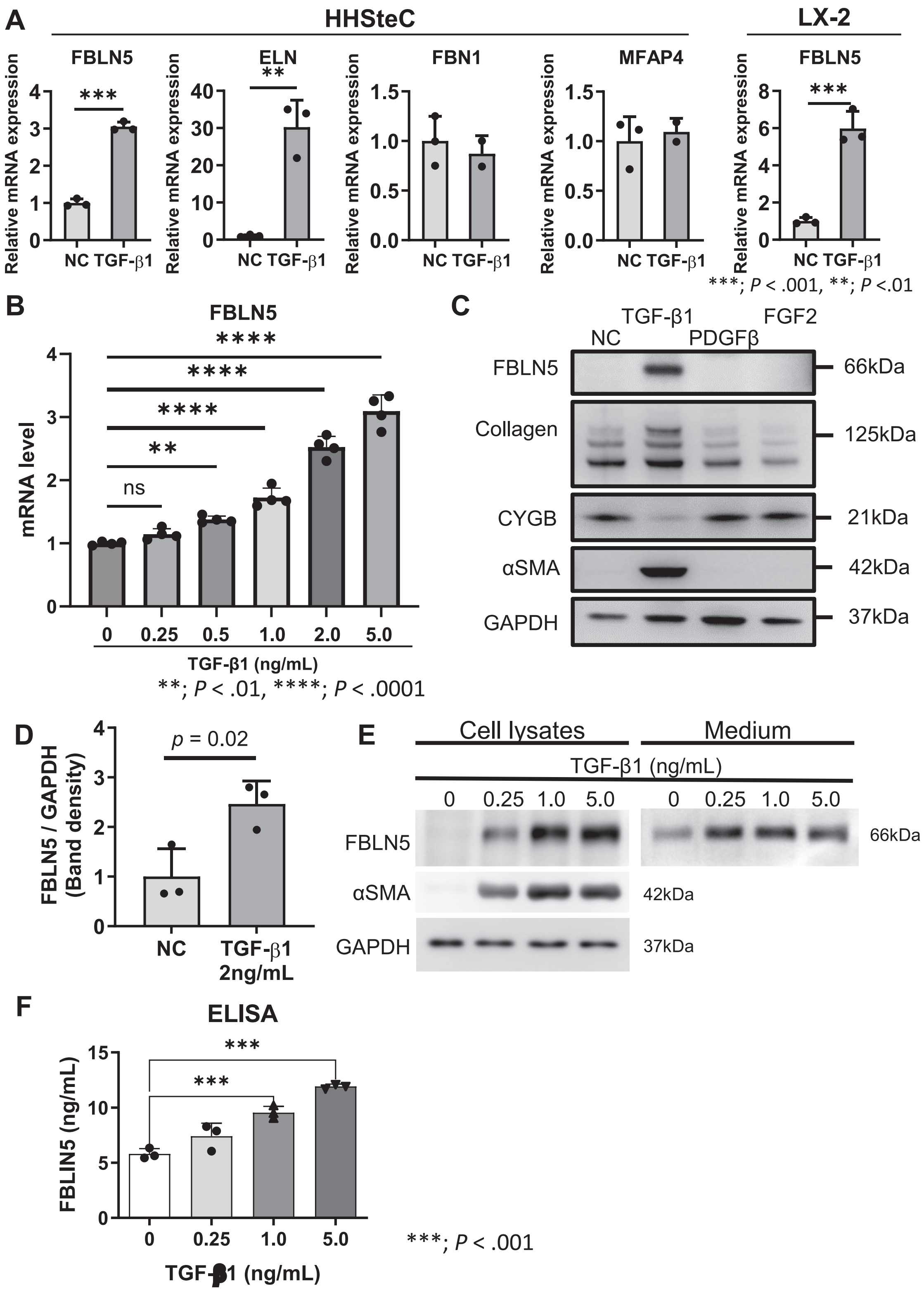

Figure 3. TGF-β1 induces FBLN5 expression in human HSCs. Elastin and FBLN5 are significantly upregulated in response to TGF-β1 in human hepatic stellate cells (HHSteC) (A). FBLN5 responds to TGF-β1 in a dose-dependent manner (B). The effect of TGF-β1 on protein expression assessed in HHSteC indicates FBLN5 is significantly increased in response to TGF-β1 along with profibrogenic markers such as α-smooth muscle actin (αSMA) or collagen, which is the opposite reaction to cytoglobin, a quiescent marker of hepatic stellate cells (C and D). This response is not observed for other growth factors, including platelet-derived growth factor (PDGF) and fibroblast growth factor (FGF) 2 (C). In addition to cell lysates, FBLN5 expression increases in the culture medium in response to TGF-β1 (E), which is confirmed using ELISA (F). CYGB, cytoglobin; ELN, elastin; GAPDH, glyceraldehyde 3-phosphate dehydrogenase; MFAP4, microfibril-associated glycoprotein 4; NC, negative control.

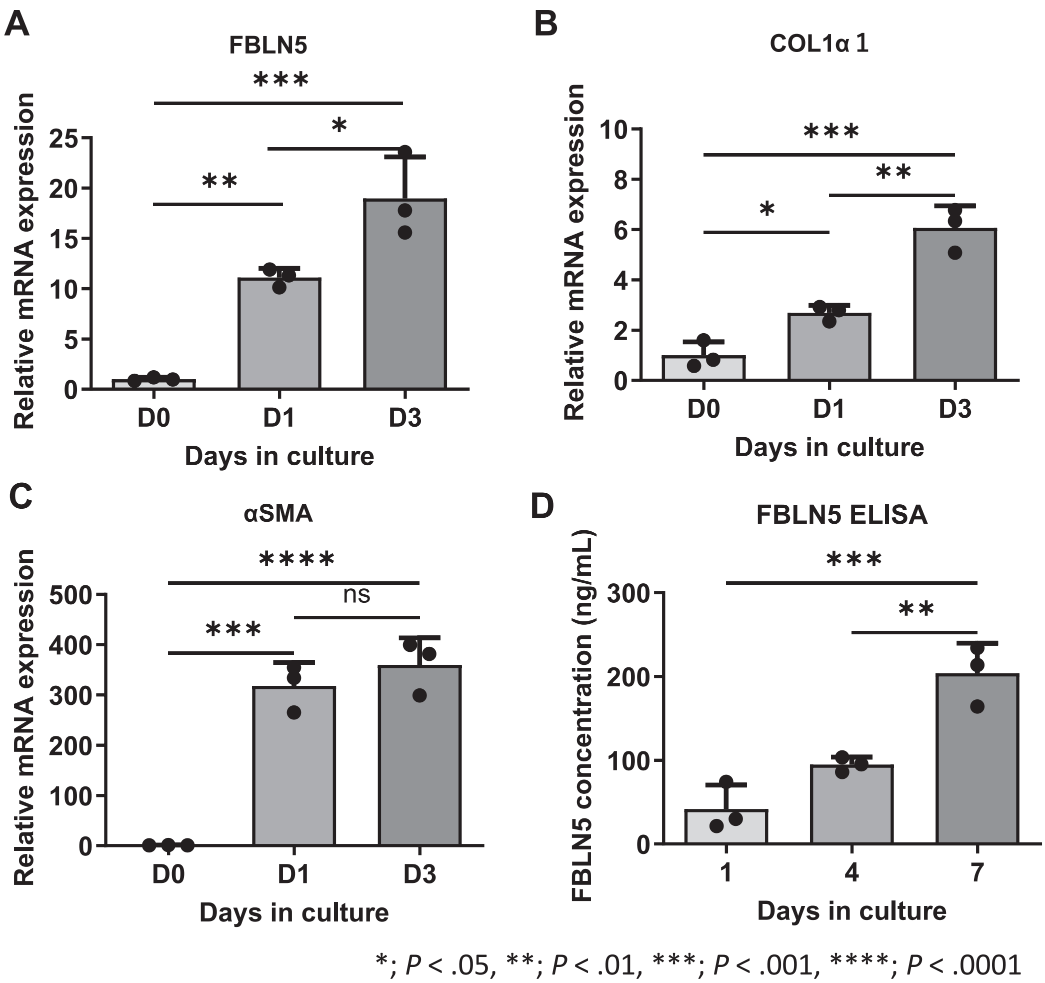

Figure 4. Increased expression of FBLN5 in spontaneously activated mouse primary HSCs *in vitro* FBLN5 upregulation is confirmed in primary mouse HSCs cultured in a plastic dish (A). The expression of collagen (B) and αSMA (C) is upregulated in primary mouse HSCs on day 1 and day 3, which indicate activation in the plastic dish. The concentration of FBLN5 in the culture medium of this model increases in a time-dependent manner (D).

shown). We further analyzed the value of plasma FBLN5 in predicting advanced fibrosis (F3 or F4) and cirrhosis (F4) using receiver-operating characteristic curves. FBLN5 showed significant predictability both for the prediction of advanced fibrosis (area under the receiver-operating characteristic [AUROC] curve: 0.845) and cirrhosis (AUROC curve: 0.823) (Figure 5C and D). Next, we compared FBLN5 with AST to platelet ratio index (APRI), FIB-4 index, and type IV collagen, a widely used marker of fibrosis. Plasma FBLN5 was significantly correlated with FIB-4 (Spearman $r = 0.533$, $P < .001$), as well as APRI ($r = 0.489$; $P < .001$). Serum collagen type IV was also significantly correlated with fibrosis stage (Figure 5B). The diagnostic performance of plasma FBLN5 for predicting F3–4 or F4 was comparable to that of the FIB-4 index, APRI, and collagen type IV (Figure 5C and D). Using the Youden index, the optimal cutoff value for FBLN5 was determined to be 1773 ng/mL. Patients with FBLN5 levels above this cutoff had a significantly higher prevalence of F3–4 fibrosis stage (81% vs 14%; $P < .001$) and F4 fibrosis stage (63% vs 10%, $P < .001$). Regression coefficients from the logistic model were used to calculate a predictive score for each patient by multiplying each marker's value by its corresponding coefficient and summing the results. The score combining FBLN5 and collagen type IV demonstrated significantly superior predictive performance compared with collagen type IV alone, as shown by the AUROC curve (Figure 5E), highlighting the additional value of FBLN5 in combination with collagen type IV. Although the score calculated from FBLN5 and the FIB-4 index showed a high AUROC of 0.894 for predicting F3–4 stage, this was not significantly greater than that of the FIB-4 index alone. In the multivariable logistic regression analyses, both FBLN5 (odds ratio 2.3 per 100 ng/mL; 95% CI: 1.4–3.9; $P = .001$) and collagen type IV (odds ratio 1.4 per 100 ng/mL; 95% CI: 1.1–1.7; $P = .004$) were significant predictors of advanced fibrosis (F3–4).

Discussion

In the present study, we demonstrated the potential of plasma FBLN5 as a predictive marker for advanced fibrosis.

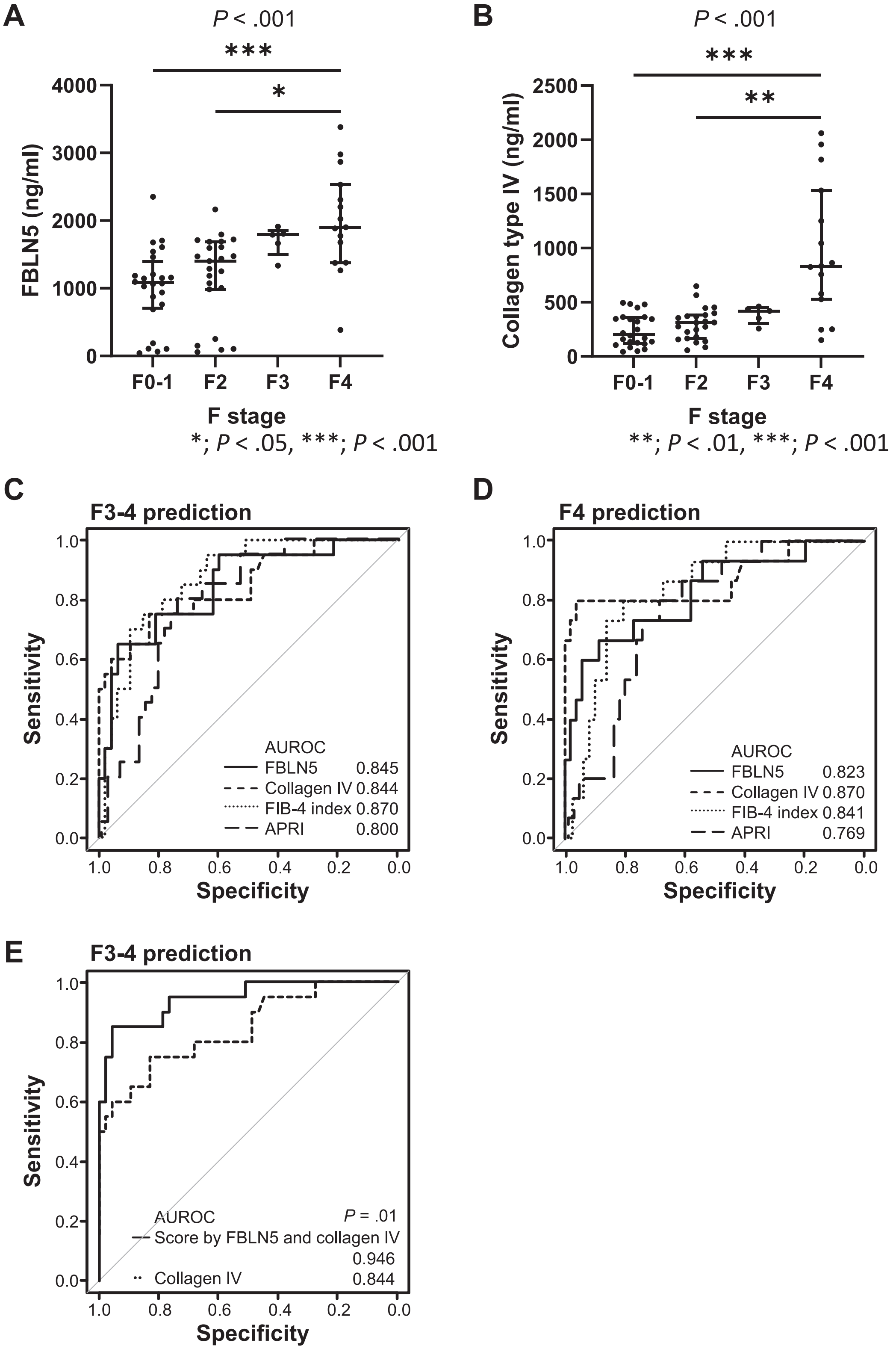

Figure 5. Plasma FBLN5 is a good predictor of progressive fibrosis in patients with hepatitis C. Plasma FBLN5 levels in patients with chronic hepatitis C significantly correlate with fibrosis stage (A). A similar trend is observed between collagen type IV, a known fibrosis marker, and fibrosis stage (B). Receiver-operating characteristic analysis reveals that plasma FBLN5 levels have a significant value for predicting advanced fibrosis (C) and cirrhosis (D). A predictive score calculated by FBLN5 and collagen type IV showed significantly better prediction compared to collagen type IV alone (E).

In liver biopsy samples obtained from patients with chronic hepatitis C, FBLN5 was coexpressed with elastic fiber and αSMA, an activated HSC marker, and its expression correlated significantly with the stage of liver fibrosis. snRNA-seq showed predominant FBLN5 expression in the HSC/fibroblast cluster, with higher levels in cirrhotic livers. Furthermore, in vitro studies showed that FBLN5 is overexpressed and secreted from activated human HSCs in response to TGF-β1. This was also confirmed in spontaneously activated mouse primary HSCs. Finally, we found that peripheral plasma FBLN5 levels were predictive of advanced fibrosis, suggesting its potential utility as a noninvasive marker for liver fibrosis.

FBLN5 is a member of the fibulin family, which has an integrin-binding motif and epidermal growth factor-like modules. During the embryonic period, FBLN5 plays an important role in arterial wall development. In a previous study, FBLN5 knockout mice express tortuous aortas; however, no abnormalities have been reported in the liver.[22] In adults, FBLN5 is expressed less in the vessels; however, once an injury occurs in these vessels, FBLN5 is re-expressed.[25,34] In the liver, FBLN5 is expressed during portal fibrosis induced by idiopathic portal hypertension.[35] A comprehensive microarray analysis that compared mild (F0-1) and severe (F3–4) steatotic liver disease showed that FBLN5 was upregulated in severe steatotic liver disease along with collagen species.[36] Moreover, a proteomic analysis revealed that the protein and gene expression of FBLN5 in the liver is significantly correlated with the severity of fibrosis.[27] Our findings are consistent with these studies, showing that HSCs activated by TGF-β1 produced FBLN5, highlighting the role of TGF-β as a strong activator of human HSCs and a main contributor in liver fibrosis progression.[37] Previous research by Schiemann et al also supports our results showing that FBLN5 is induced by TGF-β through a Smad3-independent pathway in the murine fibroblast.[38] Given above, our results expand on existing knowledge by identifying plasma FBLN5 as a potential biomarker for liver fibrosis. Although FBLN5 may represent a potential therapeutic target for liver fibrosis, safety concerns arise from data showing that FBLN5 knockout mice develop lethal vascular complications during embryogenesis. Thus, liver-specific strategies or other selective approaches would be necessary to consider FBLN5 as a feasible therapeutic target.

Our previous study reported that the fibrosis stage did not improve shortly after successful treatment of chronic hepatitis C, whereas the activity grade showed significant improvement following antiviral therapy.[39] Moreover, histological improvement after treatment is an independent predictor of hepatocellular carcinoma development.[40] Several studies, including ours, have suggested that elastic fibers are abundant in advanced-stage fibrosis[20,41] and that patients with a higher amount of elastic fibers are at higher risk of hepatocellular carcinoma development[10] or adverse outcomes if they have advanced fibrosis.[11] Therefore, the amount of elastic fibers and FBLN5 in the liver provides critical information for assessing both the regression of fibrosis and the clinical course of patients with advanced fibrosis. As fibrolytic therapies continue to be developed, it is crucial to identify appropriate candidates and establish reliable markers to evaluate fibrosis regression. Our data suggest that FBLN5 could be an indicator for determining the need for antifibrotic treatment. When comparing plasma FBLN5 with collagen type IV, a well-known marker of liver fibrosis, FBLN5 showed an additive value for predicting advanced fibrosis or cirrhosis. Although the correlation between plasma FBLN5 and fibrosis stage was modest ($r = 0.30$), this may be due to the ordinal nature of fibrosis staging (0–4) and the nonlinear increase in FBLN5, with marked elevation observed mainly in F4, reflecting advanced elastic fiber accumulation. Further studies are necessary to explore how FBLN5 can be to integrate other noninvasive fibrosis markers. In addition, it should also be noted that plasma FBLN5 levels are more indicative of liver elastic fibers than collagen fibers which is its unique role in fibrosis evaluation.

The present study had several limitations. The major limitations of this study are its retrospective design and small sample size. These results should be validated by larger prospective cohort studies. Another limitation is that we could not compare the value of plasma FBLN5 to that of any noninvasive imaging elastography, which is becoming a standard technique for estimating the grade of liver fibrosis. In addition, the utility of FBLN5 should be investigated in liver fibrosis with etiologies other than hepatitis C. While we conducted snRNA-seq analysis to confirm upregulation of FBLN5 in steatotic liver disease, more comprehensive studies are needed. Lastly, the number of cases that we can assess both IHC scoring of FBLN5 and plasma FBLN5 were limited, making it difficult to perform the reliable correlation analysis.

In conclusion, we demonstrated that plasma FBLN5, a component of elastic fibers, is a novel marker of liver fibrosis in patients with chronic hepatitis C. Liver FBLN5 colocalized with αSMA-positive HSCs, and in vitro experimentation confirmed that FBLN5 was upregulated in activated HSCs and secreted into cultured medium. Therefore, we conclude that plasma FBLN5 has the potential to identify liver fibrosis, particularly in relation to activated HSCs.

Supplementary Materials

Material associated with this article can be found in the online version at https://doi.org/10.1016/j.gastha.2025.100827.

References

1. Martinello M, Solomon SS, Terrault NA, et al. Hepatitis C. Lancet 2023;402:1085–1096.
2. Polaris Observatory HCVC. Global change in hepatitis C virus prevalence and cascade of care between 2015 and 2020: a modelling study. Lancet Gastroenterol Hepatol 2022;7:396–415.

3. Stanaway JD, Flaxman AD, Naghavi M, et al. The global burden of viral hepatitis from 1990 to 2013: findings from the Global Burden of Disease Study 2013. Lancet 2016;388:1081–1088.
4. Friedman SL, Bansal MB. Reversal of hepatic fibrosis – fact or fantasy? Hepatology 2006;43:S82–S88.
5. Brenner DA. Reversibility of liver fibrosis. Gastroenterol Hepatol (N Y) 2013;9:737–739.
6. Friedman SL. Clarity and challenges in tissue fibrosis. In: Nakao K, Minato N, Uemoto S, eds. Innovative medicine. Tokyo: Basic Research and Development, 2015:187–194.
7. Sun M, Kisseleva T. Reversibility of liver fibrosis. Clin Res Hepatol Gastroenterol 2015;39(Suppl 1):S60–S63.
8. Issa R, Zhou X, Constandinou CM, et al. Spontaneous recovery from micronodular cirrhosis: evidence for incomplete resolution associated with matrix cross-linking. Gastroenterology 2004;126:1795–1808.
9. Chen W, Yan X, Xu A, et al. Dynamics of elastin in liver fibrosis: accumulates late during progression and degrades slowly in regression. J Cell Physiol 2019;234:22613–22622.
10. Yasui Y, Abe T, Kurosaki M, et al. Elastin Fiber accumulation in liver correlates with the development of hepatocellular carcinoma. PLoS One 2016;11:e0154558.
11. Kendall TJ, Dolman GE, Duff CM, et al. Hepatic elastin content is predictive of adverse outcome in advanced fibrotic liver disease. Histopathology 2018;73:90–100.
12. Wai CT, Greenson JK, Fontana RJ, et al. A simple noninvasive index can predict both significant fibrosis and cirrhosis in patients with chronic hepatitis C. Hepatology 2003;38:518–526.
13. Rosenberg WM, Voelker M, Thiel R, et al. Serum markers detect the presence of liver fibrosis: a cohort study. Gastroenterology 2004;127:1704–1713.
14. Sterling RK, Lissen E, Clumeck N, et al. Development of a simple noninvasive index to predict significant fibrosis in patients with HIV/HCV coinfection. Hepatology 2006;43:1317–1325.
15. Toshima T, Shirabe K, Ikegami T, et al. A novel serum marker, glycosylated Wisteria floribunda agglutinin-positive Mac-2 binding protein (WFA(+)-M2BP), for assessing liver fibrosis. J Gastroenterol 2015;50:76–84.
16. Afdhal NH, Bacon BR, Patel K, et al. Accuracy of fibroscan, compared with histology, in analysis of liver fibrosis in patients with hepatitis B or C: a United States multicenter study. Clin Gastroenterol Hepatol 2015;13:772–779.e1–3.
17. Hu X, Qiu L, Liu D, et al. Acoustic Radiation Force Impulse (ARFI) Elastography for noninvasive evaluation of hepatic fibrosis in chronic hepatitis B and C patients: a systematic review and meta-analysis. Med Ultrason 2017;19:23–31.
18. Huwart L, Sempoux C, Vicaut E, et al. Magnetic resonance elastography for the noninvasive staging of liver fibrosis. Gastroenterology 2008;135:32–40.
19. Nakayama H, Itoh H, Kunita S, et al. Presence of perivenular elastic fibers in nonalcoholic steatohepatitis Fibrosis Stage III. Histol Histopathol 2008;23:407–409.
20. Masugi Y, Abe T, Tsujikawa H, et al. Quantitative assessment of liver fibrosis reveals a nonlinear association with fibrosis stage in nonalcoholic fatty liver disease. Hepatol Commun 2018;2:58–68.
21. Shin SJ, Yanagisawa H. Recent updates on the molecular network of elastic fiber formation. Essays Biochem 2019;63:365–376.
22. Nakamura T, Lozano PR, Ikeda Y, et al. Fibulin-5/DANCE is essential for elastogenesis in vivo. Nature 2002;415:171–175.
23. Yanagisawa H, Davis EC, Starcher BC, et al. Fibulin-5 is an elastin-binding protein essential for elastic fibre development in vivo. Nature 2002;415:168–171.
24. Hirai M, Ohbayashi T, Horiguchi M, et al. Fibulin-5/DANCE has an elastogenic organizer activity that is abrogated by proteolytic cleavage in vivo. J Cell Biol 2007;176:1061–1071.
25. Nakamura T, Ruiz-Lozano P, Lindner V, et al. DANCE, a novel secreted RGD protein expressed in developing, atherosclerotic, and balloon-injured arteries. J Biol Chem 1999;274:22476–22483.
26. Timpl R, Sasaki T, Kostka G, et al. Fibulins: a versatile family of extracellular matrix proteins. Nat Rev Mol Cell Biol 2003;4:479–489.
27. Bracht T, Schweinsberg V, Trippler M, et al. Analysis of disease-associated protein expression using quantitative proteomics-fibulin-5 is expressed in association with hepatic fibrosis. J Proteome Res 2015;14:2278–2286.
28. Intraobserver and interobserver variations in liver biopsy interpretation in patients with chronic hepatitis C. The French METAVIR Cooperative Study Group. Hepatology 1994;20:15–20.
29. Bedossa P, Poynard T. An algorithm for the grading of activity in chronic hepatitis C. The METAVIR Cooperative Study Group. Hepatology 1996;24:289–293.
30. Sato-Matsubara M, Matsubara T, Daikoku A, et al. Fibroblast growth factor 2 (FGF2) regulates cytoglobin expression and activation of human hepatic stellate cells via JNK signaling. J Biol Chem 2017;292:18961–18972.
31. Okina Y, Sato-Matsubara M, Matsubara T, et al. TGF-beta1-driven reduction of cytoglobin leads to oxidative DNA damage in stellate cells during non-alcoholic steatohepatitis. J Hepatol 2020;73:882–895.
32. Kanda Y. Investigation of the freely available easy-to-use software 'EZR' for medical statistics. Bone Marrow Transpl 2013;48:452–458.
33. Koui Y, Himeno M, Mori Y, et al. Development of human iPSC-derived quiescent hepatic stellate cell-like cells for drug discovery and in vitro disease modeling. Stem Cell Rep 2021;16:3050–3063.
34. Spencer JA, Hacker SL, Davis EC, et al. Altered vascular remodeling in fibulin-5-deficient mice reveals a role of fibulin-5 in smooth muscle cell proliferation and migration. Proc Natl Acad Sci U S A 2005;102:2946–2951.
35. Sato Y, Sawada S, Nakanuma Y. Fibulin-5 is involved in phlebosclerosis of major portal vein branches associated with elastic fiber deposition in idiopathic portal hypertension. Hepatol Res 2008;38:166–173.
36. Moylan CA, Pang H, Dellinger A, et al. Hepatic gene expression profiles differentiate presymptomatic patients with mild versus severe nonalcoholic fatty liver disease. Hepatology 2014;59:471–482.
37. Tsuchida T, Friedman SL. Mechanisms of hepatic stellate cell activation. Nat Rev Gastroenterol Hepatol 2017;14:397–411.

38. Schiemann WP, Blobe GC, Kalume DE, et al. Context-specific effects of fibulin-5 (DANCE/EVEC) on cell proliferation, motility, and invasion. Fibulin-5 is induced by transforming growth factor-beta and affects protein kinase cascades. J Biol Chem 2002;277:27367–27377.
39. Enomoto M, Ikura Y, Tamori A, et al. Short-term histological evaluations after achieving a sustained virologic response to direct-acting antiviral treatment for chronic hepatitis C. United Eur Gastroenterol J 2018;6:1391–1400.
40. Motoyama H, Tamori A, Kubo S, et al. Stagnation of histopathological improvement is a predictor of hepatocellular carcinoma development after hepatitis C virus eradication. PLoS One 2018;13:e0194163.
41. Yasui Y, Abe T, Kurosaki M, et al. Non-invasive liver fibrosis assessment correlates with collagen and elastic fiber quantity in patients with hepatitis C virus infection. Hepatol Res 2019;49:33–41.

Received November 20, 2024. Accepted September 30, 2025.

Correspondence:
Address correspondence to: Norifumi Kawada, MD, PhD, Department of Hepatology, Graduate School of Medicine, Osaka Metropolitan University, Graduate School of Medicine, 1-4-3, Asahi-machi, Abeno-ku, Osaka 545-8585, Japan. e-mail: kawadanori@omu.ac.jp.

Acknowledgments:
The authors express their sincere thanks to all patients and their families who participated in this study. In vitro analyses were performed in the Research Support Platform of Osaka Metropolitan University Graduate School of Medicine; the authors thank all platform technicians for their support. YY was supported by the Uehara Memorial Foundation Overseas Research Fellowship. The authors thank all the members of the Department of Anatomy and Regenerative Biology and the Department of Hepatology at Osaka Metropolitan University for their meaningful comments on this study.

Authors' Contributions:
Conceptualization: Yutaka Yasui, Misako Sato-Matsubara. Data curation: Yutaka Yasui, Masaru Enomoto, Tsutomu Matsubara. Formal analysis: Yutaka Yasui, Misako Sato-Matsubara. Funding acquisition: Misako Sato-Matsubara, Norifumi Kawada. Investigation: Yutaka Yasui, Misako Sato-Matsubara, Truong Huu Hoang, Atsuko Daikoku, Hideto Yuasa, Hideki Fujii, Yoshihiro Ikura, Etsushi Kawamura, Sawako Uchida-Kobayashi. Methodology: Misako Sato-Matsubara, Tsutomu Matsubara, Norifumi Kawada. Project administration: Misako Sato-Matsubara, Norifumi Kawada. Resources: Misako Sato-Matsubara, Norifumi Kawada. Supervision: Norifumi Kawada. Validation: Misako Sato-Matsubara, Atsuko Daikoku. Visualization: Yutaka Yasui, Misako Sato-Matsubara. Writing – original draft: Yutaka Yasui, Misako Sato-Matsubara. Writing – review and editing: Masaru Enomoto, Sawako Uchida-Kobayashi, Akihiro Tamori, Norifumi Kawada.

Conflicts of Interest:
The authors disclose no conflicts.

Funding:
This study was supported by a grant-in-aid from the Japan Agency for Medical Research and Development (grant number: 23fk0210107, URL: http://www.amed.go.jp/en/). The funding source was not involved in the study design, data collection, analysis, and interpretation, or manuscript writing.

Ethical Statement:
All study participants provided informed consent, and the study design was approved by the Ethics Committee of Osaka Metropolitan University Graduate School of Medicine (approval number 1646).

Data Transparency Statement:
Study materials will be made available upon request. The snRNA-seq analysis was carried out with data set GSE185477, GSE174748, and GSE212046.

Reporting Guidelines:
STROBE.

Gastro Hep Advances, Volume 5

Supplemental information

Plasma Fibulin-5 as a Novel Marker for Advanced Fibrosis in Chronic Hepatitis C

Yutaka Yasui, Misako Sato-Matsubara, Masaru Enomoto, Tsutomu Matsubara, Mana Kosugi, Kirara Inoue, Truong Huu Hoang, Hideto Yuasa, Hideki Fujii, Atsuko Daikoku, Yoshihiro Ikura, Etsushi Kawamura, Sawako Uchida-Kobayashi, Akihiro Tamori, and Norifumi Kawada

Supplementary Material & methods

Plasma fibulin-5 as a novel marker for advanced fibrosis in chronic hepatitis C

Yutaka Yasui, Misako Sato-Matsubara, Masaru Enomoto, Tsutomu Matsubara, Mana Kosugi, Kirara Inoue, Truong Huu Hoang, Hideto Yuasa, Hideki Fujii, Atsuko Daikoku, Yoshihiro Ikura, Etsushi Kawamura, Sawako Uchida-Kobayashi, Akihiro Tamori, Norifumi Kawada

Table of Contents

Fig. S1 Study flowchart

IHC: immunohistochemistry

Fig. S2 Fibulin-5 (FBLN5)-positive areas were significantly correlated with fibers quantified by azan staining and elastic fibers quantified by orcein staining

Fig. S3 The protein expression of human hepatic stellate cell (HHSteC) and human dermal fibroblast in response to transforming growth factor (TGF) β-1.

Fig. S4 The HHSteC culture medium was analyzed to confirm whether FBLN5 was secreted from activated HSCs. The Coomassie Brilliant Blue staining confirmed that the amount of protein in the medium samples was consistent across all samples, indicating equal protein loading.

Table S1. The list of primers for real-time PCR

Table S2. Multivariate analysis for the factors associated with F3–F4 stage

Table S1. The list of primers for real-time PCR

Gene	Forward (5'→3')	Reverse (5'→3')
h18S	AGTCCCTGCCCTTTGTACACA	CGATCCGAGGGCCTCACTA
hELN	GCAGGAGTTAAGCCCAAGG	TGTAGGGCAGTCCATAGCCA
hFBLN5	CTCACTGTTACCATTCTGGCTC	GACTGGCGATCCAGGTCAAAG
hFBN1	TTTAGCGTCCTACACGAGCC	CCATCCAGGGCAACAGTAAGC
hMFAP4	TACCAGTCAGACGGCGTGTA	CCACTCGCAGCTCATACTTCT

PCR, polymerase chain reaction

Table S2. Multivariate analysis for the factors associated with F3–F4 stage

	Univariate analysis			Multivariate analysis		
Factors	Odds ratio	95% C.I.	P value	Odds ratio	95% C.I.	P value
Age; per 1-year increase	1.05	0.99–1.11	0.10			
Sex; male	3.44	1.12–10.5	0.03			
Platelet; per 1×10^4(/μl) increase	0.78	0.67–0.90	0.0009			
AST; per 1 (U/L) increase	1.01	1.00–1.01	0.06			
ALT; per 1 (U/L) increase	1.01	0.99–1.01	0.11			
Collagen type IV; per 100 (ng/ml) increase	1.92	1.31–2.81	0.0008	1.37	1.11–1.70	0.004
FBLN5; per 100 (ng/ml) increase	1.28	1.11–1.48	0.0007	2.33	1.41–3.88	0.001

Fig. S1

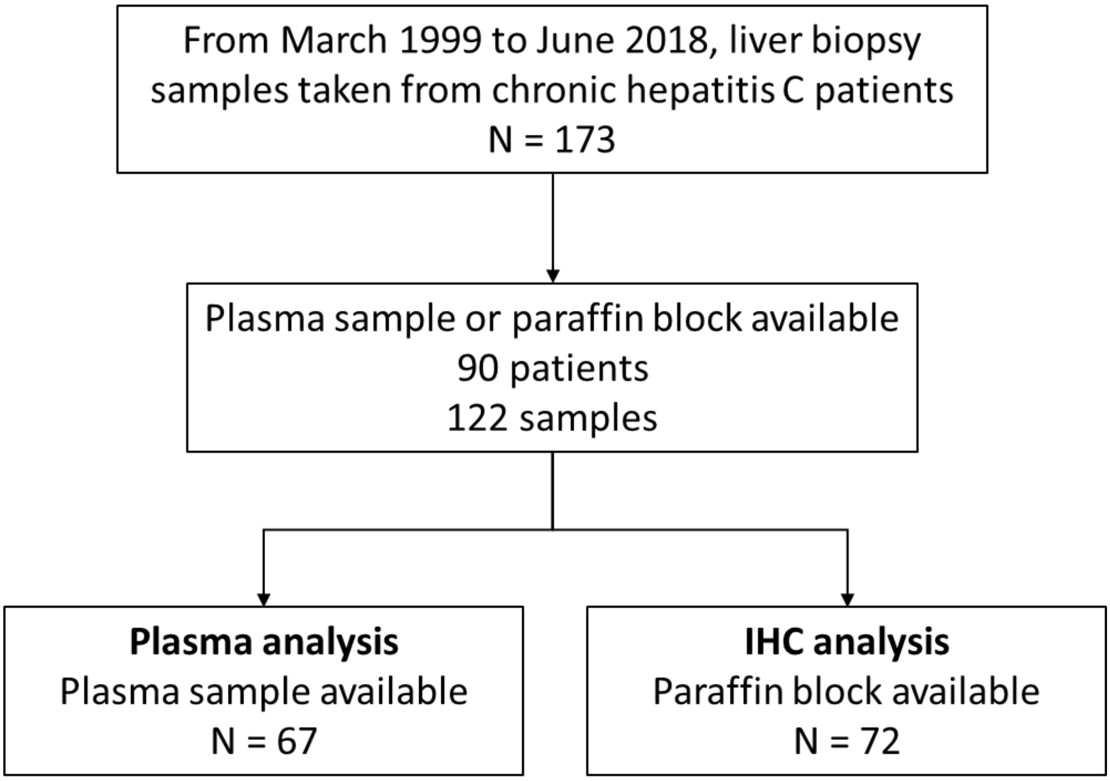

33

Fig. S2

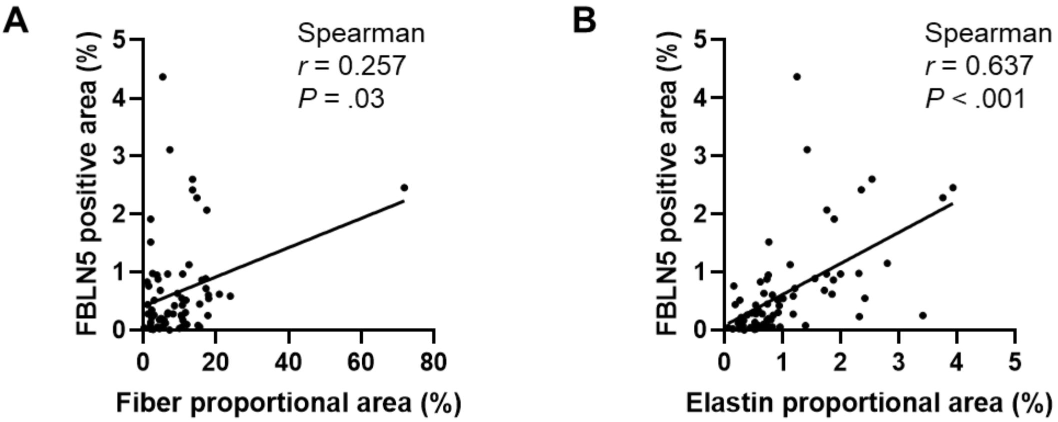

Fig. S3

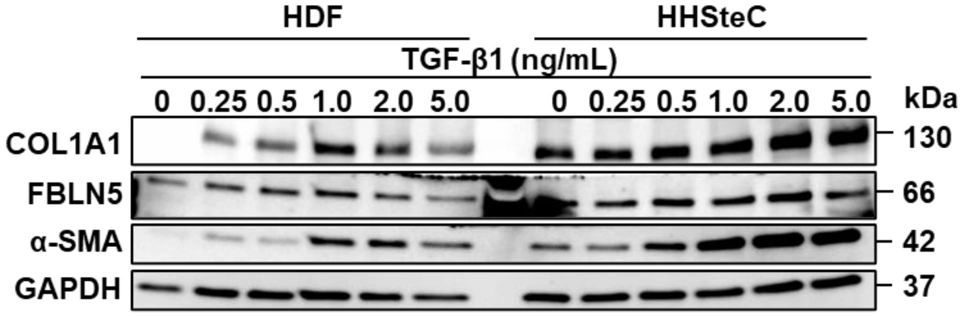

34

Fig. S4

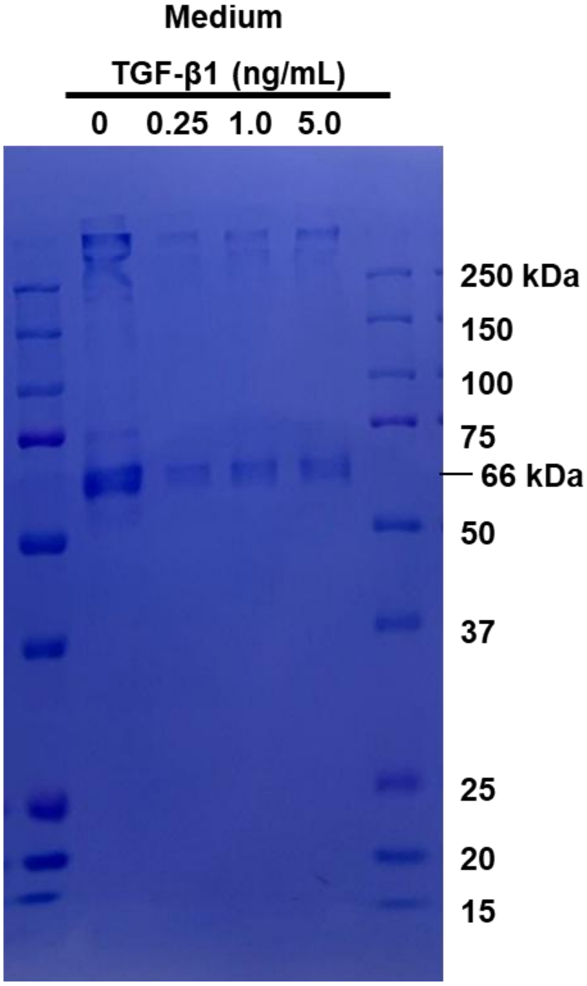

Supplement: Extended pdf [file mmc2.pdf]
